# Accuracy on Scanned Images of Full Arch Models with Orthodontic Brackets by Various Intraoral Scanners in the Presence of Artificial Saliva

**DOI:** 10.1155/2020/2920804

**Published:** 2020-02-27

**Authors:** Jihu Song, Minji Kim

**Affiliations:** Department of Orthodontics, College of Medicine, Ewha Womans University, Seoul, Republic of Korea

## Abstract

**Aim:**

This study aims to evaluate the accuracy of scanned images of 4 clinically used intraoral scanners (CS3600, i500, Trios3, Omnicam) when scanning the surface of full arch models with various kinds of orthodontic brackets in the presence of artificial saliva. *Materials and Methods*. Four study models were prepared; bonded with ceramic, metal, and resin brackets, respectively, and without brackets. Reference images were taken by scanning the models with an industrial scanner. Study models were then applied with an artificial saliva and scanned 10 times, respectively, with the above 4 intraoral scanners. All images were converted to STL file format and analyzed with 3D analysis software. By superimposing with the reference images, mean maximum discrepancy values and mean discrepancy values were collected and compared. For statistical analysis, two-way ANOVA was used.

**Results:**

Omnicam (1.247 ± 0.255) showed higher mean maximum discrepancy values. CS3600 (0.758 ± 0.170), Trios3 (0.854 ± 0.166), and i500 (0.975 ± 0.172) performed relatively favourably. Resin (1.119 ± 0.255) and metal (1.086 ± 0.132) brackets showed higher mean maximum discrepancy values. Nonbracket (0.776 ± 0.250) and ceramic bracket (0.853 ± 0.269) models generally showed lower mean maximum discrepancy values in studied scanners. In mean discrepancy values, the difference between scanners was not statistically significant whereas among brackets, resin bracketed models (0.093 ± 0.142) showed the highest value.

**Conclusion:**

Intraoral scanners and brackets had significant influences on the scanned images with application of artificial saliva on the study models. It may be expected to have similar outcomes in an intraoral environment. Some data showed the discrepancy values up to about 1.5 mm that would require more caution in using intraoral scanners for production of detailed appliances and records.

## 1. Introduction

The clinical usage of intraoral scanners has become more and more common. For the last decade, the effectiveness of intraoral scanners was studied to prove their practicability in various clinical settings [1–9]. Digital impression systems, when proving their credibility to replace conventional impression methods will benefit the orthodontic clinicians and patients. It has been shown that patients are generally satisfied and prefer recordings by intraoral scanners over conventional alginate impression technique [10]. They will help to save physical storage spaces and time since the treatment process requires multiple times of impression taking. Conventional impression technique results in more discomfort especially for the orthodontic patients due to the presence of brackets and wire and additional time and effort required because of that.

The scope of researches on intraoral scanning has been limited mostly to a prosthetic point of view [1, 3–9]. However, orthodontic conditions such as brackets and wires start to be included in more recent studies [11–15]. It is only that impression taking during orthodontic treatments involves several limitations due to intraoral conditions. Contamination of saliva on the teeth and the presence of various kinds of brackets is one of the intraoral conditions that may have direct impact on the accuracy of the scanned image [16, 17]. There was a study on the effect of water on the surface of dental restorative materials [18]. Design of the study, however, was only about the effect of reflection of water film on the material margin imbedded in a single tooth. Comparison between in vivo and extraoral model scanning was done in 2013, yet it was limited to one specific intraoral scanner. It was not under orthodontic treatment conditions; it was not full arch study nor did it involve brackets [19]. There have been many changes and developments in the technology since then [2].

In the area of orthodontic treatment, full arch scanning is rather important and useful. As mentioned above, orthodontic treatment involves multiple times of full arch impression. With increased tooth mobility during the treatment process, intraoral scanning, if proven more competent, would lessen patients discomfort which otherwise would have been caused by conventional alginate impression technique. Therefore, a research on full arch scanning was designed to verify the practicability of various intraoral scanners in orthodontic treatment conditions. Artificial saliva was applied on bracket-bonded study models to simulate the wet condition in the mouth. The null hypothesis was that there would be no significant difference between the reference and the variables.

## 2. Materials and Methods

### 2.1. Study Model Fabrication

**Figure 1:**
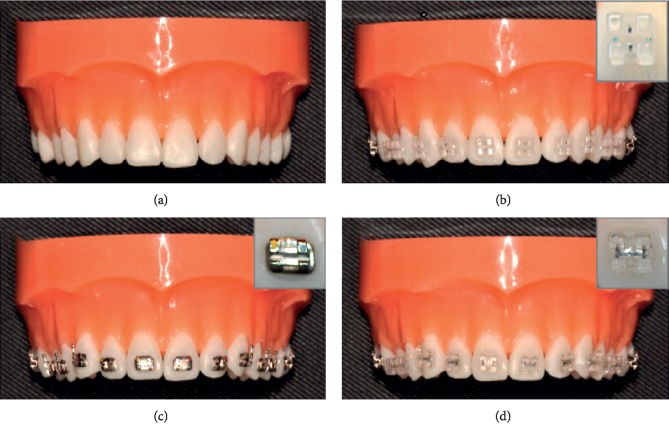
Study models and brackets. (a). Nonbracket model (b). Ceramic bracket model (c). Metal bracket model. (d). Resin bracket model.

Four upper dental study models (dentiform, Tomy Inc., Fuchushi, Japan) were set up as follows as they would be in clinical settings (Figure 1): a study model without any brackets and with ceramic brackets (Crystaline® ceramic bracket, Tomy Inc., Fuchushi, Japan), metal brackets (Micro arch® Appliances, Tomy Inc., Fuchushi, Japan), and resin brackets (Ortho Esther MB, Tomy Inc., Fuchushi, Japan) bonded, respectively, to all 12 teeth from upper right first molar to left molar of each study model. 3M Transbond™ XT Light Cure Adhesive system (3M, Monrovia, U.S.A.) was used as bonding agents. Brackets were located in their ideal positions on the facial axis of clinical crown.

### 2.2. Reference Scanned Data

Four kinds of prepared study models were scanned with stereoScan R8 S-075 (AICON 3D systems, Biberweg, Braunschweig; industrial scanner with error range ±10 um). These scanned data were used as reference data for each category. The procedure was handled by an expert in the area.

### 2.3. Application of Artificial Saliva

To simulate the oral environment of orthodontic patients (i.e., contamination by saliva with presence of brackets), clinically used artificial saliva (Biotène® Moisturizing Spray, GSK Consumer Healthcare, Warren, USA) was applied to the tooth surfaces and brackets with an art brush, three pumps for each application. More artificial saliva was applied before the moisture dried up. Two to three applications for each set of scanning (10-time scan for each scanner) were necessary.

### 2.4. Four Different Intraoral Scanners Used for Scanning of Each Study Model

**Figure 2:**
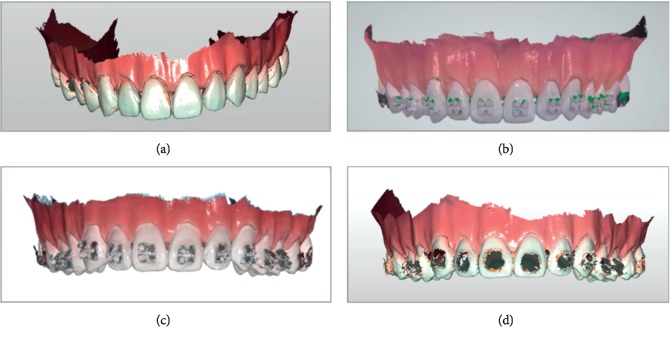
Scanned image examples; scanned by intraoral scanners. (a) Nonbracket/cs3600. (b) Ceramic brackets/Trios3, (c) Metal brackets/i500. (d) Resin brackets/cs3600.

Each study model was then scanned 10 times repeatedly by 4 intraoral scanners: CS3600 (Carestream Dental, Atlanta, USA), i500 (Medit Corp., Seoul, Korea), Trios3 (3Shape, Copenhagen, Denmark), and Omnicam (Sirona, Bensheim, Germany) (Figure 2). About 3 minutes of break was given after each scan. The experimenter was trained for given intraoral scanners with dozens of scan practices [20]. The operating software was CS3600 6.14.7.3 for CS3600, MeditiScan 1.1.1.1 for i500, and Trios 2015-1 1.4.7.5 and Omnicamcerec software 4.5 for Omnicam. The scanning sequence started from the buccoocclusal surface of the left molars, passing towards the anterior teeth performing an s-shaped movement capturing the buccal, incisal, and lingual surfaces in the anterior segment, and progressing to the buccoocclusal surface of the right molars, returning via the lingual surfaces to the left molars where the scanning started. Additional scans were done for missed spots.

### 2.5. Datasets

All datasets were converted to STL (Standard Triangulated Language [8], Standard Tessellation Language [19], and Stereolithography [21]) file format via manufacturers' certified software for standardization. Four datasets of 4 study models were taken from the reference scanner as reference data. Each study model was scanned 10 times by each intraoral scanner with application of artificial saliva. As a result, 160 intraoral scanner datasets were produced in this study.

### 2.6. Superimposition Assessment

**Figure 3:**
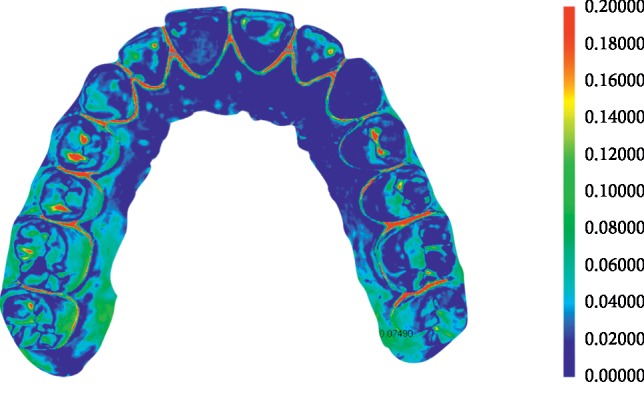
Superimposed image of reference and scanned image.

Image data were analyzed with the 3-dimensional analysis software (Geomagic Verify™, Geomagic, Morrisville, USA). Each image was trimmed right below (about 1 mm) the dentogingival line before superimposition process to ease the analysis by minimizing the size of the data and to exclude obvious optical artifacts of unimportant areas [13]. It is also the range that most orthodontic treatments require for analysis and device production. Four reference data (dry; stereoScan R8 S-075 (AICON 3D systems, Biberweg, Braunschweig) were then superimposed with the images of respective intraoral scanners (Figure 3). The best-fit algorithm of the analysis software was applied for comparison. Positive and negative discrepancies were shown by the software as in a coloured map [22]. The discrepancy values were calculated from RMS (Root Mean Square) values of 10 superimposition data of each scanner.

Colour range was set as 0 to 0.2 mm. Two images of nonbracket study model scanned by the reference and CS3600 scanner were aligned by best-fit algorithm. The result was shown as a colour map. The program also calculates mean discrepancy values and mean maximum discrepancy values. Redder and bluer colours indicate more discrepancy. Geomagic Verify™ was used.

## 3. Results

Ten superimpositions were performed for each category. Reference data were superimposed with 10 datasets of nonbracket study model images scanned by CS3600. The superimposition program gives both mean discrepancy values and mean maximum discrepancy values. The average of maximum deviation values for the category was termed ‘Mean maximum discrepancy value'. The average of mean deviation values throughout two-image 3D comparisons was termed as ‘mean discrepancy value'. The deviation was between two images scanned by the reference scanner and an intraoral scanner.

### 3.1. Mean Maximum Discrepancy values (Unit: mm)

**Figure 4:**
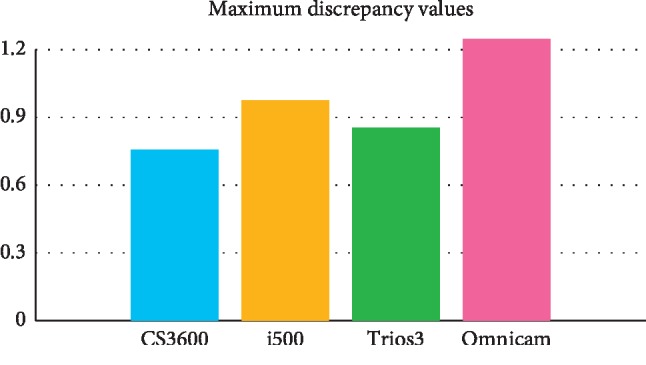
Discrepancies in superimposition of reference and scanned images within scanners; the average maximum discrepancy of the superimposed data of two images.

**Figure 5:**
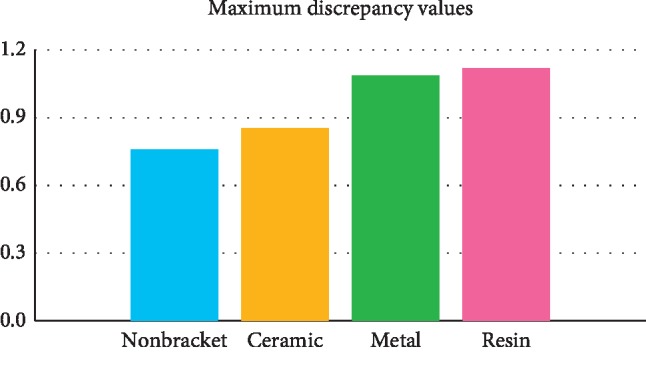
Discrepancies in superimposition of reference and scanned image within brackets; the average maximum discrepancy of the superimposed data of two images.

Two-Way ANOVA was performed to verify the scanner and bracket's impact on ‘Mean maximum discrepancy values' of deviations of images scanned by intraoral scanners (Table 1). Both scanners (*p* < 0.05) and brackets (*p* < 0.05) had a statistically significant impact. The interaction term between scanners and brackets was statistically significant (*p* < 0.05) (Figures 4 and 5).

The influence by the brackets was observed. In CS3600, the mean maximum discrepancy values according to each kind of study model were ordered as follows: metal (0.96 ± .04)>resin (0.86 ± .04)>ceramic (0.61 ± .08) = nonbracket (0.60 ± .08) (*p* < 0.05). In Trios3, they were ordered as follows: resin (1.05 ± 0.05)>metal (0.97 ± 0.01)>ceramic (0.75 ± .06)>nonbracket (0.65 ± 0.03) (*p* < 0.05).

As studying the influence of scanners on ‘Mean maximum discrepancy values' within brackets, the following were noted. The mean maximum discrepancy values for the metal bracketed study models were ordered as follows: Omnicam (1.26 ± 0.03)>i500 (1.15 ± 0.06)>Trios3 (0.97 ± 0.01) = CS3600 (0.96 ± 0.04) (*p* < 0.05). In resin bracketed study models, the value of images scanned by Omnicam was also the highest (1.49 ± 0.13), followed by i500 (1.08 ± 0.15)/Trios3 (1.05 ± 0.05) and CS3600 (0.86 ± 0.04) (*p* < 0.05).

### 3.2. Mean Discrepancy values (Unit: mm)

Two-Way ANOVA was performed to verify the scanner and bracket's impact on ‘mean discrepancy values' of deviations of images scanned by intraoral scanners. It was determined that the influence of scanners on mean discrepancy values was not statistically significant. However, brackets had a statistically significant influence on mean discrepancy values (*p* < 0.05).

In CS3600, the mean discrepancy values of the brackets were ordered as follows: resin (0.074 ± 0.00) = nonbracket (0.063 ± 0.015) = ceramic (0.062 ± 0.014)>metal (0.019 ± 0.008) (*p* < 0.05). In Trios3, the mean discrepancy values depending on the study models were ordered as follows: resin (0.081 ± 0.007)>ceramic (0.061 ± 0.011) = nonbracket (0.056 ± 0.006)>metal (0.007 ± 0.007) (*p* < 0.05).

In regard to the influence of scanners within brackets, the mean discrepancy values for the metal bracketed study model were ordered as follows: i500 (0.028 ± 0.008)>CS3600 (0.019 ± 0.008)>Trios3 (0.007 ± 0.007) = Omnicam (0.006 ± 0.009) (*p* < 0.05). In ceramic bracketed model, the mean discrepancy values of CS3600 and Trios3 (0.061 ± 0.011) were the highest (0.062 ± .014), followed by i500 (0.040 ± 0.008)/Omnicam (0.027 ± 0.009) (*p* < 0.05).

## 4. Discussion

The objective of this study was to evaluate the accuracy of scanned images of 4 clinically used intraoral scanners when scanning the surface of full arch models with various kinds of orthodontic brackets in the presence of artificial saliva.

3D superimposition analysis (Geomagic Verify™) shows and calculates maximum discrepancy values as well as mean discrepancy values. The RMS values of 10 superimposed 3D images were collected and averaged for each scanner and bracket entry in this study. This represents the total absolute amount of discrepancies. Mean discrepancy values are the average of discrepancy values between the reference and the scanned images by respective intraoral scanners throughout the 3D comparison whereas mean maximum discrepancy values indicate the maximum discrepancy of the superimposed data of two images [1]. They were termed as mean discrepancy values and mean maximum discrepancy values, respectively. Overall mean discrepancy values may appear small, yet, at some areas, there may be outstandingly or irregularly large values. This constitutes mean maximum discrepancy values. When maximum deviation appears at important parts of the image, it may imply lower quality of the scanned images and may cause clinical flaws. Mean maximum discrepancy values, therefore, were more looked into and analyzed with clinical interests.

Within brackets, Omnicam showed the highest maximum discrepancy values in most study models (*p* < 0.05). This corresponds with results of other studies including partial and full arch research [16, 23]. On the other hand, there was a study result of having no significant difference under in vivo condition between Omnicam and Trios3 [24]. CS3600 showed generally higher accuracy among scanners and it was often observed in other studies as well [16, 23]. However, as long as scanners are concerned, there have been several different results that no one specific intraoral scanner could be named as the best [14, 20].

As the impacts within scanners were considered, the mean maximum discrepancy values in CS3600 appeared in the order of metal > resin > ceramic = nonbracketed study model. In Trios3, it was the order of resin > metal > ceramic > nonbracketed study model. Both results were statistically significant. Nonbracketed study model showed the lowest mean maximum discrepancy values with statistical significance in all 4 intraoral scanners: the presence of brackets certainly caused more discrepancies to the result. The mean maximum discrepancy values of resin and metal bracketed models were on the higher side. All showed statistically significant differences with an exception of the data from Omnicam. Translucency of the material may have contributed to such result [18, 21]. Kurz et al. in his experiment in 2015 showed that resin material had higher discrepancy [18]. According to Li et al., higher translucency objects resulted in lower scanning accuracy [21]. This coincides with the result of this study.

There were some observations to consider when comparing the scanned images; resin brackets were not scanned in full. Therefore, the comparisons between the scanners could not be decisive (Figure 2). Most images of the resin brackets were later processed and completed by the software. Resin bracketed study model images produced the highest mean maximum discrepancy values and mean discrepancy values in most scanners. Ceramic brackets seem translucent yet they did not present high discrepancy values comparably. This may be because the polycrystalline ceramic bracket used in this study does not reflect much light by its nature. If monocrystalline ceramic was used, the result may have been different.

Metal brackets were not easily scanned and more shots had to be taken in order to finish the scan. However, the image was more complete than resin brackets (Figure 2). This may also have contributed to the fact that metal bracketed model images had lower mean values. that is, more shots had to be taken to complete each scan, thus less differences between scans in total. Lower discrepancy values are preferable in clinical use and its accuracy. It should be reminded, however, that after certain number of shots, most intraoral scanners' lag time gets longer or rescan image prompt pops up which results in longer scanning time. It seems that 3D production may not be a reliable option yet for brackets or other orthodontic devices made of resin or with minor details.

Scanners demonstrated similar mean discrepancy values. However, mean maximum discrepancy values are clinically significant. CS3600 and Trios 3 demonstrated favourable results in most bracket models. Omnicam showed higher mean maximum discrepancy values than most scanners. This was not much different from the results of several previous studies [16, 23]. The value was more than 1 mm in some study models which could be critical depending on the location. However, one can reason this with the presence of artificial saliva. Water film thickness on the materials such as ceramics, metals, and composites can be as thick as 1600 *um.* [18] The experimenter tried to apply the artificial saliva as even as possible. However, there could be more human error in it during that specific session. It may be expected also that the updated software (Omnicam 4.6) may draw better results.

There were some bubbles formed by the artificial saliva (Biotène®) captured as engraved concave images rather than embossed convex images as they actually formed.

**Figure 6:**
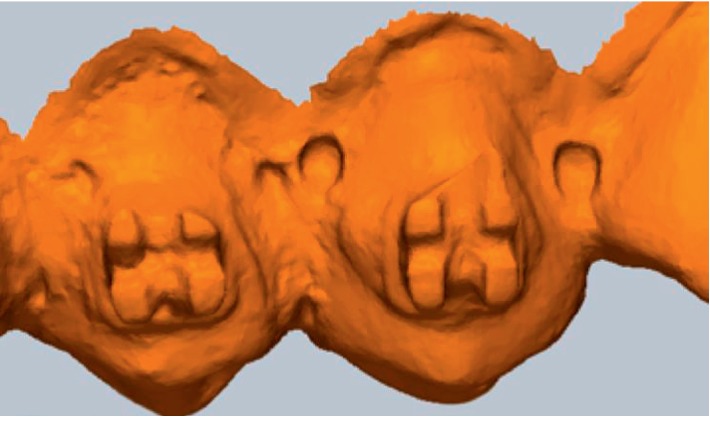
Bubbles formed on the surface of study models; the image captured on Geomagic Verify™ before superimposition. The shape is concave rather than convex as seen in bubbles.

The scanners probably recognize and capture only the inner half of the bubble (Figure 6). How much discrepancies the formed bubbles may have caused could not be estimated. However, it is certain that it would also contribute to the sum of discrepancies.

There are several other aspects of oral environment other than contamination of saliva: temperature, humidity, interruption from soft tissues, limited spaces, and so on. The study models used in this experiment having a different degree of surface reflection from real teeth may also have limited the result [23]. However, this may be excused since intraoral situations would include various kinds of dental prostheses such as gold, ceramic, and resin to scan. Flügge et al. drew a conclusion in 2013 that intraoral condition (saliva, limited spacing) contributed to the inaccuracy of a scan. [19] There have been significant developments in intraoral scanner technology and its design that they overcame many limitations since then [25]. There have been numerous studies since 2015 on the accuracy of intraoral scanner images in various prosthetic and orthodontic conditions [11, 26–31]. However, most of them were with dry models. In vivo studies should be performed to support and prove reliability and practicability of intraoral scanners in full.

Scanned images by the industrial scanner without application of artificial saliva were used as reference data. For clinical purposes such as production of appliances or taking digital records, it is the real images of teeth and brackets, in orthodontics, that are required regardless of the presence of saliva in the mouth. Thus such condition was given for the reference data. Whether application of artificial saliva directly and proportionally affected the values of discrepancies could not be determined. To be clear in this regard, a different design of study would be necessary.

The introduction of powder-free 3D intraoral scanners in 2006 was welcomed by clinicians and patients for its convenience, accuracy, and freeing from unpleasantness in the mouth [18]. However, one may consider spraying the power partially on the metal and resin surface of the intraoral objects such as brackets and other dental prostheses to improve the result and efficiency of the procedure [27].

It was found that there were many terms used to indicate discrepancies in previous studies: mean values and mean maximum values [1], mean-maximum [16], average-maximum [20], and so on. As more extensive studies need to be done to fully utilize intraoral scanners in various areas of clinical and academic dentistry, it would be beneficial to unify admixed terms in order.

Oral environment involves other aspects as well that contamination of saliva is not the sole element. In vivo study for intraoral scanners to verify its practicability and impacts by various conditions could be carried out for further research.

When artificial saliva was applied, it had statistically significant influences on the scanned images of bracketed and nonbracketed full arch models. It may be expected to have similar outcomes in an intraoral environment with saliva. Some discrepancy values were regarded as large. When intraoral scanners are to be used in production of detailed appliance and records, more consideration may be given with that regard.

## 5. Conclusion

In this study, four different kinds of orthodontic models (nonbrackets and ceramic/metal/resin brackets) were applied with artificial saliva and scanned with 4 clinically used intraoral scanners (CS3600, i500, Trios3, and Omnicam). Each image was superimposed with the reference image and the impact of saliva was analyzed.

Models with brackets with more translucent and/or more reflective of lights such as resin and metal brackets tended to show higher discrepancy values [18]. Among the scanners studied, CS3600 and Trios3 yielded more accurate results. The values were significantly different yet generally acceptable for the digital systems to be used clinically considering the fact that conventional methods do result in the range of discrepancies as well [20]. However, some data showed the discrepancy values up to about 1.5 mm that more caution would be required for production of detailed appliances and records.

## Figures and Tables

**Table 1:** Discrepancy results from superimposition of reference and scanned images (mm).

	Bracket (B) *Mea*n ± std. dev							*p* value	(A) × (B)
	Scanner (A)	Nonbracket	Ceramic	Metal	Resin	Average values	Scanner	Bracket	
Max	CS3600	0.60 ± 0.08^c^_B_	0.61 ± 0.08^c^_C_	0.96 ± 0.04^a^_C_	0.86 ± 0.04^b^_C_	0.758 ± 0.170_C_		<0.001^*∗*^	
	i500	0.82 ± 0.05^b^_AB_	0.85 ± 0.08^b^_BC_	1.15 ± 0.06^a^_B_	1.08 ± 0.15^a^_B_	0.975 ± 0.172_B_		<0.001^*∗*^	
	Trios3	0.65 ± 0.03^d^_B_	0.75 ± 0.06^c^_BC_	0.97 ± 0.01^b^_C_	1.05 ± 0.05^a^_B_	0.854 ± 0.166_C_		<0.001^*∗*^	
	Omnicam	1.03 ± 0.37^b^_A_	1.20 ± 0.29^ab^_A_	1.26 ± 0.03^ab^_A_	1.49 ± 0.13^a^_A_	1.247 ± 0.286_A_		0.002^*∗*^	
	*p* value	<0.001^*∗*^	<0.001^*∗*^	<0.001^*∗*^	<0.001^*∗*^	<0.001^*∗*^	<0.001^*∗*^	<0.001^*∗*^	0.010
	Average	0.776 ± 0.250^b^	0.853 ± 0.269^a^	1.086 ± 0.132^b^	1.119 ± 0.255^a^			<0.001^*∗*^	

Mean	CS3600	0.063 ± 0.015^a^	0.062 ± 0.014^a^_A_	0.019 ± 0.008^b^_B_	0.074 ± 0.008^a^_A_	0.055 ± 0.024		<0.001^*∗*^	
	i500	0.036 ± 0.005^bc^	0.040 ± 0.008^ab^_B_	0.028 ± 0.008^c^_A_	0.046 ± 0.009^a^_B_	0.037 ± 0.152		<0.001^*∗*^	
	Trios3	0.056 ± 0.006^b^	0.061 ± 0.011^b^_A_	0.007 ± 0.007^c^_C_	0.081 ± 0.007^a^_A_	0.051 ± 0.029		<0.001^*∗*^	
	Omnicam	0.008 ± 0.017^b^	0.027 ± 0.009^ab^_B_	0.006 ± 0.009^b^_C_	0.079 ± 0.015^a^_A_	0.030 ± 0.064		0.031^*∗*^	
	*p* value	0.172	<0.001^*∗*^	<0.001^*∗*^	<0.001^*∗*^	0.579	<0.001^*∗*^	<0.001^*∗*^	0.507
	Average	0.041 ± 0.061^bc^	0.048 ± 0.019^ab^	0.015 ± 0.018^c^	0.070 ± 0.142^a^			<0.001^*∗*^	

*p* < 0.05:^*∗*^ Max: mean maximum discrepancy values. The maximum discrepancy of the superimposed data of two images. Mean: mean discrepancy values. The average of discrepancy values throughout the 3D comparison. 2-Way ANOVA was used for statistical analysis. a >b > c > d:superscript lowercase letters indicate statistically significant differences between brackets. A > B > C > D: subscript uppercase letters indicate statistically significant differences between scanners.

## Data Availability

The STL data used to support the findings of this study are available from the corresponding author upon request.
